# Adult 10-year survivors after liver transplantation: a single-institution experience over 40 years

**DOI:** 10.1007/s13304-023-01598-1

**Published:** 2023-07-27

**Authors:** Quirino Lai, Gianluca Mennini, Stefano Ginanni Corradini, Flaminia Ferri, Stefano Fonte, Francesco Pugliese, Manuela Merli, Massimo Rossi

**Affiliations:** 1 General Surgery and Organ Transplantation Unit, Department of General and Specialty Surgery, Sapienza University of Rome, AOU Umberto I Policlinico of Rome, Viale del Policlinico 155, 00161 Rome, Italy; 2 Department of Translational and Precision Medicine, Sapienza University of Rome, AOU Umberto I Policlinico of Rome, Rome, Italy; 3 Department of Anesthesiology and Critical Care, University of Rome Sapienza, AOU Umberto I Policlinico of Rome, Rome, Italy

**Keywords:** Donor age, Recipient age, Liver function, Patient survival, Graft survival

## Abstract

Liver transplantation (LT) represents the best cure for several acute and chronic liver diseases. Several studies reported excellent mid-term survivals after LT. However, lesser evidence has been reported on very long (10- and 20-year) follow-up results. This study aims to analyze the monocentric LT experience of the Sapienza University of Rome to identify the pre-operatively available parameters limiting a 10-year post-transplant survival. A total of 491 patients transplanted between 1982 and 2012 were enrolled. The cohort was split into two groups, namely the Short Surviving Group (< 10 years; n = 228, 46.4%) and the Long Surviving Group (≥ 10 years; n = 263, 53.6%). Several differences were reported between the two groups regarding initial liver function, surgical techniques adopted, and immunosuppression. Four variables emerged as statistically relevant as independent risk factors for not reaching at least 10 years of follow-up: recipient age (OR = 1.02; P = 0.01), donor age (OR = 1.01; P = 0.03), being transplanted during the eighties (OR = 6.46; P < 0.0001) and nineties (OR = 2.63; P < 0.0001), and the UNOS status 1-2A (OR = 2.62; P < 0.0001). LT confirms to be an extraordinary therapy for several severe liver diseases, consenting to reach in half of the transplanted cases even more than 20 years of follow-up. The initial liver function and the donor and recipient ages are relevant in impacting long-term survival after transplantation. A broad commitment from many professional groups, including surgeons, hepatologists, and anesthesiologists, is necessary. The achievement of excellent results in terms of long-term survival is proof of the effectiveness of this multidisciplinary collaboration.

## Introduction

Liver transplantation (LT) represents the gold-standard therapy for several acute and chronic liver diseases [1] and different primitive and secondary hepatic tumors [2]. Prof. Starzl performed the first LT procedure in 1963 in Denver, Colorado [3]. However, LT was considered an experimental procedure until 1983, when the American National Institute of Health Consensus Development Conference recognized it as a standard therapy for curing liver diseases [4].

Starting from this period, the number of LT national programs grew exponentially, with an incredible spread of LT procedures worldwide [5]. Among these programs, the Italian one started in May 1982 at Sapienza University of Rome. Extraordinary innovations have also been observed in the field of transplantation, with a growing ability in liver disease management, immunosuppression, surgical techniques, and anesthesiological care [6–8].

Similarly, the long-term results after LT have shown a progressive increase [5]. From the initially conventionally accepted post-LT 5-year patient survival of 50% [9], the recently reported survivals have reached the 5- and 10-year rates of 70–75% and 50–60%, respectively [5, 10].

There have been numerous reports on survival outcomes after LT with short- and mid-term follow-up. However, only a few studies are available for 10- or 20-year long-term survivors [11, 12].

This study aims to analyze the monocentric LT experience of the Sapienza University of Rome, with the intent a) to compare a group of patients surviving more than ten years from LT (Long Surviving Group) with a group of patients unable to reach this follow-up length (Short Surviving Group), and b) to identify the parameters limiting a long post-transplant surviving.

## Methods

### Study design

The present study is a retrospective monocentric research based on a prospectively maintained database of patients transplanted in the Azienda Ospedaliero-Universitaria Policlinico Umberto I of Rome, Sapienza University of Rome, Italy. This study followed the Strengthening the Reporting of Observational Studies in Epidemiology (STROBE) reporting guidelines. The institutional review board of Azienda Ospedaliero-Universitaria Policlinico Umberto I approved the study.

### Setting

Participants included the patients undergoing liver transplantation in the General Surgery and Organ Transplantation Unit of the Azienda Ospedaliero-Universitaria Policlinico Umberto I of Rome, Sapienza University of Rome, Italy.

### Population

Seven hundred and seventy-seven patients consecutively received 818 LT from May 1982 to May 2022. All the patients transplanted during this period were initially considered for the study. Exclusion criteria were: (a) donation after circulatory death (n = 1), (b) living donation (n = 9), (c) combined transplantation (n = 18; liver-kidney = 12, cluster = 4, liver-hearth = 1, liver-pancreas = 1), (d) recipient age < 18 years (n = 17), (e) LT performed after May 2012 (n = 209), and (f) LT performed before May 2012 but lost at follow-up (n = 32). Four hundred and ninety-one patients were finally enrolled for the present study.

### Variables and data collection

Data collected in the study included:

- recipient characteristics = age, sex, period of transplant (1982–1991, 1992–2001, 2002–2012), blood group, Caucasian ethnicity, United Network for Organ Sharing (UNOS) status, HCC, HCV, HBV, HDV, alcohol, non-alcoholic steatohepatitis (NASH) or cryptogenic, biliary cirrhosis, acute liver failure, other liver diseases.
- Donor characteristics = age, sex, blood group, donor-recipient blood iso-group, donor-recipient sex match.
- Transplantation characteristics = split liver, type of perfusion liquid, total ischemia time, type of caval reconstruction.
- Immunosuppression characteristics = induction drugs (steroids, immunoglobulins), maintenance therapy (cyclosporine, tacrolimus, mammalian target of rapamycin [mTor] inhibitor, azathioprine, derivate of mycophenolic acid).

### Definitions

According to the UNOS status, the definitions were [13]:

- status 1 = patient with fulminant liver failure;
- status 2A = patient hospitalized in the hospital critical care unit due to chronic liver failure;
- 2B = patient with Child–Pugh score ≥ 10 or ≥ 7 + at least one criterion among unresponsive active variceal hemorrhage, hepato-renal syndrome, spontaneous bacterial peritonitis, and refractory ascites;
- 3 = patient requiring continuous medical care but followed at home or near the transplant center.

Primary-non-function (PNF) was defined as the need for retransplantation or death within seven days of LT without any other defined cause. Primary dysfunction (PDF) was defined as the need for retransplantation or death after seven days of LT without any other defined cause.

Patient death was defined as any transplant-related or unrelated event of death observed at any time from LT. Patient death time was calculated as the time from LT to the death event during the follow-up. Patients alive at the last follow-up were censored. The later follow-up date was May 31, 2022.

### Statistical analysis

Baseline characteristics of each data set were presented as medians and interquartile ranges (IQR) for continuous variables and as numbers and percentages for discrete variables. Comparisons between groups were made using Fisher’s exact test or the chi-square test for categorical variables, as appropriate. Mann–Whitney was used for continuous variables. No missing data relative to study covariates used for constructing the model were observed; therefore, no data interpolation was required.

A multivariable logistic regression model was constructed to identify the variables connected with a poor ability to reach a survival of at least ten years after transplant. The variables for creating the model were preliminarily selected using a Least Absolute Shrinkage and Selection Operator (LASSO) regression (stepwise regression with backward elimination). Twenty-three different variables were initially tested: recipient age, donor-recipient blood iso-group, donor-recipient sex match, Caucasian ethnicity, UNOS status 1-2A, period of transplant (1982–1991 vs. 1992–2001 vs. 2002–2012; the latter period as reference), split liver, HCC, HCV, HBV, HBV + HDV, alcohol, biliary cirrhosis, acute liver failure, caval reconstruction technique, total ischemia time, donor age, induction with steroids, induction with immunoglobulin, cyclosporine, tacrolimus, azathioprine, derivatives of mycophenolic acid. Odds ratio (OR) and 95% confidence intervals (95.0% CI) were reported.

Survival curves were performed using the Kaplan–Meier method. Log-rank test was used to compare the survival results.

A P value < 0.05 was considered statistically significant. Statistical analyses were conducted using SPSS 27.0 (SPSS Inc., Chicago, IL, USA).

## Results

The median follow-up period for the entire cohort (N = 491) was 10.4 years (IQR = 0.7–15.6). The cohort was split into two groups, namely the Short Surviving (SS) Group (n = 228, 46.4%) and the Long Surviving (LS) Group (n = 263, 53.6%). In the LS Group, the median period of follow-up reached 15.2 years (IQR = 12.3–19.7). In detail, 134/491 (27.3%) and 56/491 (11.4%) had a follow-up meeting or exceeding the 15 and 20 years from LT, respectively. The most long-lived patient reached 33.3 years of follow-up.

### Comparison between long- and short-term survivors

Recipient-, donor- and transplant-related characteristics of the patients composing the two groups are reported in Table 1. Some differences were observed comparing the two groups. In detail, the LS Group was composed of patients more commonly transplanted in a later period (2002–2012 vs. 1982–1991, P < 0.0001), and with a smaller percentage of cases transplanted in UNOS status 1 or 2A (17.5 vs. 31.6%, P < 0.0001). As for the donor characteristics, it was less common in the LS Group to observe cases transplanted using donors with blood iso-group compatibility (4.9 vs. 101%, P = 0.04) and with an F–M sex match (25.1 vs. 28.9%, P = 0.04). The long survivors had a shorter ischemia time (median 460 vs. 490 min, P < 0.0001) and less common use of total caval replacement with veno-venous bypass as the technique of caval reconstruction (35.0 vs. 28.2%, P = 0.005).

**Table 1:** Recipient-, donor- and transplant-related characteristics in the two groups

Variables	Short surviving group (228, 46.4%)	Long surviving group (n = 263, 53.6%)	P value
	N (%) or median (IQR)		
Recipient			
Age, years	52 (43–59)	52 (43–59)	1.00
Sex (F/M)	74 (32.5)/154 (67.5)	69 (26.2)/194 (73.8)	0.14
Period of transplant			
1982–1991	35 (15.4)	17 (6.5)	< 0.0001
1992–2001	104 (45.6)	101 (38.4)	
2002–2012	89 (39.0)	145 (55.1)	
Blood group			
O	99 (43.4)	104 (39.5)	
A	93 (40.8)	106 (40.3)	0.48
B	30 (13.2)	40 (15.2)	
AB	6 (2.6)	13 (4.9)	
Caucasian ethnicity	225 (98.7)	258 (98.1)	0.73
UNOS status			
1	13 (5.7)	12 (4.6)	
2A	59 (25.9)	34 (12.9)	< 0.0001
2B	150 (65.8)	192 (73.0)	
3	6 (2.6)	25 (9.5)	
HCC*	72 (31.6)	83 (31.6)	1.00
HCV*	110 (48.2)	106 (40.3)	0.08
HBV*	62 (27.2)	67 (25.5)	0.68
+ HDV	13 (5.7)	14 (5.3)	1.00
Alcohol*	36 (15.8)	60 (22.8)	0.053
NASH or cryptogenic*	8 (3.5)	20 (7.6)	0.054
Biliary cholangiopathies*	14 (6.1)	15 (5.7)	0.85
Acute liver failure*	13 (5.7)	12 (4.6)	0.68
Other diseases*	28 (12.3)	38 (14.4)	0.51
Donor			
Age, years	38 (23–55)	38 (23–56)	0.80
Sex (F/M)	109 (47.8)/119 (52.2)	96 (36.5)/167 (63.5)	0.01
Blood group			
O	120 (52.6)	113 (43.0)	
A	81 (35.5)	103 (39.2)	0.10
B	24 (10.5)	39 (14.8)	
AB	3 (1.3)	8 (3.0)	
Donor-recipient blood iso-group	23 (10.1)	13 (4.9)	0.04
Donor-recipient sex match			
F–M	66 (28.9)	66 (25.1)	
F–F	43 (18.9)	30 (11.4)	0.04
M–F	31 (13.6)	39 (14.8)	
M–M	88 (38.6)	128 (48.7)	
Transplantation			
Split liver	0 (–)	5 (1.9)	0.06
Type of perfusion			
Collins	15 (6.6)	2 (0.8)	
Belzer	179 (78.5)	392 (81.0)	0.002
Celsior	34 (14.9)	48 (18.3)	
Total ischemia time, min	490 (433–604)	460 (390–540)	< 0.0001
Type of caval reconstruction			
Caval replacement	166 (72.8)	171 (65.0)	0.005
Piggyback	40 (17.5)	39 (14.8)	
Piggyback LL	22 (9.6)	53 (20.2)	

*N* number, *IQR* interquartile ranges, *F* female, *M* male, *UNOS* united network for organ sharing, *HCC* hepatocellular carcinoma, *HCV* hepatitis C virus, *HBV* hepatitis B virus, *HDV* hepatitis D virus, *NASH* non-alcoholic steato-hepatitis, *LL* latero-lateral

*Some patients had multiple causes of hepatopathy requiring transplantation

Investigating in detail the immunosuppression drugs used immediately after the LT (Table 2), it was possible to note that the long survivors less commonly used for induction anti-thymocyte globulin (14.1 vs. 21.1%, P = 0.04) and more commonly basiliximab (2.3% vs. no cases, P = 0.03). As for the maintenance therapy, calcineurin inhibitors (97.0 vs. 87.3%, P < 0.0001) and derivates of mycophenolic acid (59.3 vs. 36.8%, P < 0.0001) were more commonly used in long survivors.

**Table 2:** Initial immunosuppression characteristics in the two groups

Variables	Short surviving group (228, 46.4%)	Long surviving group (n = 263, 53.6%)	P value
	N (%) or median (IQR)		
Induction			
Steroids	220 (96.5)	256 (97.3)	0.61
Immunoglobulins (any)	71 (31.1)	64 (24.3)	0.11
Basiliximab	0 (–)	6 (2.3)	0.03
ATG	48 (21.1)	37 (14.1)	0.04
Muromonab-CD3	23 (10.1)	21 (8.0)	0.43
Maintenance therapy			
CNI*	199 (87.3)	255 (97.0)	< 0.0001
Cyclosporine	128 (56.1)	145 (55.1)	0.86
Tacrolimus	82 (36.0)	123 (46.8)	0.02
Twice daily	82 (36.0)	123 (46.8)	0.02
Once daily	2 (0.9)	14 (5.3)	0.009
mTor inhibitor	0 (–)	3 (1.1)	0.25
Azathioprine	78 (34.2)	71 (27.0)	0.09
Derivates of mycophenolic acid	84 (36.8)	156 (59.3)	< 0.0001

*N* number, *IQR* interquartile ranges, *ATG* antibodies anti-thyroglobulin, *CD* cluster of differentiation, *CNI* calcineurin inhibitor, *mTOR* mammalian target of rapamycin

*Some patients switched during the transplant hospital stay and therefore had multiple drugs concerning the conventional induction + triple maintenance therapy

### Causes of death in the transplanted patients

**Fig. 1:**
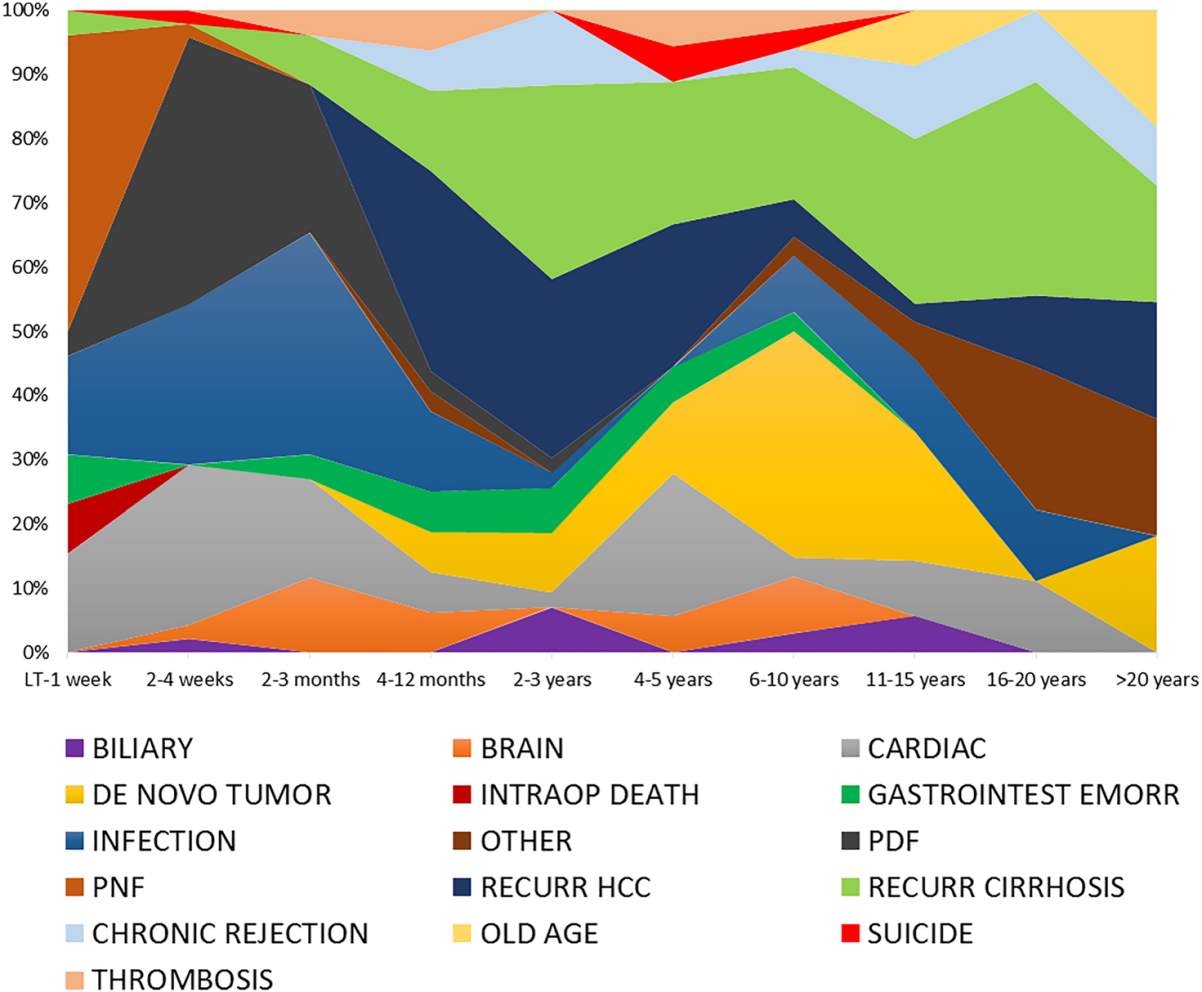
Causes of death stratified according to the time passed from LT to the death event

The investigated population observed 284/491 (57.8%) death events. Figure 1 displays the causes of death, correlating them with the time passed from LT to the death event. In the SS Group, as expected, all the cases died. In the LS Group, 56 (21.3%) deaths were reported (P < 0.0001) (Table 3). Consequently, when the different causes of death were investigated in the two groups, the short survivors almost always had more death events for each reason. In detail, early causes of death were observed exclusively in the SS Group, like intraoperative death (P = 0.10), PNF and PDF (both P < 0.0001), vascular thromboses (P = 0.02), and gastrointestinal bleeding (P = 0.0004). Also other categories of death typically correlated with more extended follow-up periods were more commonly observed in the SS Group, like infection (1.9 vs. 14.5%, P < 0.0001), cardiac (1.5 vs. 12.3%, P < 0.0001) and cerebral events (no cases vs. 4.4%, P = 0.0004), recurrence of HCC (1.5 vs. 12.3%, P < 0.0001), HCV recurrence (3.0 vs. 9.6%, P = 0.002), and de novo tumor (3.8 vs. 8.8%, P = 0.02). Only in the case of very old age as a cause of death, LS Group had more cases reported (1.9% vs. no cases, P = 0.06).

**Table 3:** Causes of patient death in the two groups

Variables	Short surviving group (228, 46.4%)	Long surviving group (n = 263, 53.6%)	P
	N (%) or median (IQR)		
Total number of deaths	228 (100.0)	56 (21.3)	< 0.0001
Infection (any type)	33 (14.5)	5 (1.9)	< 0.0001
Cardiac	28 (12.3)	4 (1.5)	< 0.0001
PDF	28 (12.3)	0 (–)	< 0.0001
Recurrence HCC	28 (12.3)	4 (1.5)	< 0.0001
Recurrence HCV	22 (9.6)	8 (3.0)	0.002
De novo tumor	20 (8.8)	10 (3.8)	0.02
PNF	14 (6.1)	0 (–)	< 0.0001
Brain	10 (4.4)	0 (–)	0.0004
Gastrointestinal hemorrhage	10 (4.4)	0 (–)	0.0004
Chronic rejection	8 (3.5)	6 (2.3)	0.43
Recurrence other disease	8 (3.5)	6 (2.3)	0.43
Biliary complication	5 (2.2)	2 (0.8)	0.26
Vascular thrombosis	5 (2.2)	0 (–)	0.02
Intraoperative death	3 (1.3)	0 (–)	0.10
Recurrence HBV	3 (1.3)	2 (0.8)	0.67
Suicide	3 (1.3)	0 (–)	0.10
Extreme old age	0 (–)	5 (1.9)	0.06
Other	2 (0.8)	6 (2.3)	0.30

*N* number, *IQR* interquartile ranges, *PDF* primary dysfunction, *HCC* hepatocellular carcinoma, *HCV* hepatitis C virus, *PNF* primary non-function, *HBV* hepatitis B virus

### Risk factors for short surviving

When the factors correlated with the risk of not reaching at least ten years of follow-up after LT were investigated (Table 4), four different variables emerged as statistically relevant. In detail, both recipient (OR = 1.02, 95.0% CI = 1.01–1.04; P = 0.01) and donor ages (OR = 1.01, 95.0% CI = 1.00–1.03; P = 0.03) were correlated with a reduced opportunity to reach at least ten years of follow-up. Similarly, being transplanted during the eighties and nineties related to increased odds of not getting a prolonged survival. Specifically, a transplant during the period 1982–1991 had an OR = 6.46 (95.0% CI = 3.05–13.68; P < 0.0001), and a transplant during the period 1992–2001 had an OR = 2.63 (95.0% CI = 1.67–4.15; P < 0.0001) (period of reference: 2002–2012).

**Table 4:** Variables correlated with the risk of not reaching at least ten years of follow-up

Variables	Beta	SE	Wald	OR	95.0% CI		P
					Lower	Upper	
UNOS status 1-2A	0.96	0.24	16.66	2.62	1.65	4.16	< 0.0001
Period 2002–2012 = Ref	–	–	–	1.00	–	–	–
Period 1982–1991	1.87	0.38	23.68	6.46	3.05	13.68	< 0.0001
Period 1992–2001	0.97	0.23	17.30	2.63	1.67	4.15	< 0.0001
Recipient age	0.02	0.01	6.18	1.02	1.01	1.04	0.01
Donor age	0.01	0.01	5.00	1.01	1.00	1.03	0.03
Constant	− 2.68	0.61	19.51	0.07	–	–	< 0.0001

Hosmer–Lemeshow Test P = 0.72

Variables initially selected for constructing the model: Recipient age, donor-recipient blood iso-group, donor-recipient sex match, Caucasian ethnicity, UNOS status 1-2A, decade of transplant, split liver, HCC, HCV, HBV, HBV + HDV, alcohol, biliary cirrhosis, ALF, caval reconstruction technique, total ischemia time, donor age, induction with steroids, induction with immunoglobulin, cyclosporine, tacrolimus, azathioprine, derivatives of mycophenolic acid

*SE* standard error, *OR* odds ratio, *CI* confidence intervals, *UNOS* United Network for Organ Sharing, *HCC* hepatocellular carcinoma, *HCV* hepatitis C virus, *HBV* hepatitis B virus, *HDV* hepatitis D virus, *ALF* acute liver failure

Lastly, being transplanted with a UNOS status 1 or 2A also correlated with a poor survival (OR = 2.62, 95.0% CI = 1.65–4.16; P < 0.0001).

### Survival curves after transplantation

**Fig. 2:**
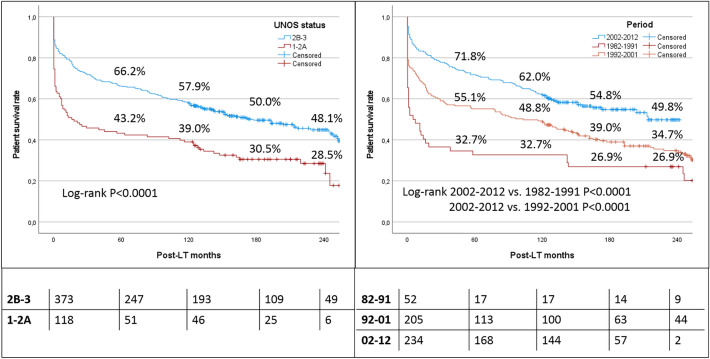
Survival curves after transplantation according to the initial UNOS status (1-2A vs. 2B-3) and the period of transplantation (1982–1991 vs. 1992–2991 vs. 2002–2012)

The overall survival rates in the entire population at 5, 10, 15, and 20 years were 60.7, 53.4, 45.0, and 40.9%, respectively. When the whole population was stratified according to the UNOS status at the time of LT, status 1-2A patients had poor survivals (5-, 10-, 15-, and 20-year rates of 43.2, 39.0, 30.5, and 28.5%, respectively). Conversely, the patients with a UNOS status 2B-3 presented excellent survivals, with rates of 66.2, 57.9, 50.0, and 48.1% at 5, 10, 15, and 20 years, respectively (log-rank P < 0.0001) (Fig. 2).

Similarly, when the population was stratified according to the period of transplantation, a progressive improvement of the results was observed from the more aged to the most recent period. In detail, 5-, 10-, 15-, and 20-year survival rates were only 32.7, 32.7, 26.9, and 26.9% in the patients transplanted during the years 1982–1991. A significant improvement was observed in the successive decade, with survival rates of 55.1, 48.8, 39.0, and 34.7% at 5, 10, 15, and 20 years after LT, respectively (log-rank P < 0.0001). Finally, the patients transplanted during the last period (i.e., 2002–2012) had the best survivals, with 5-, 10-, 15-, and 20-year rates of 71.8, 62.0, 54.8, and 49.8%, respectively (log-rank P < 0.0001) (Fig. 2).

## Discussion

Forty years ago, LT turned from an experimental procedure to a gold-standard therapy for the treatment of end-stage liver disease [4]. Several LT national programs started in the same period, comprehending the Italian one at the Sapienza University of Rome [14]. As the number of recipients has increased since the eighties, and a progressive improvement in surgical techniques, immunosuppression regimens, and infection management has been observed, the number of long-term survivors with functioning grafts has increased exponentially. Most of the previously published studies on LT typically examine short- (1-year) and medium-term (5-year) follow-ups [5, 10]. Therefore, the number of studies focused on LT surviving for more than 10 or 20 years is limited [11, 12, 15, 16]. According to the results observed in the present study, the most relevant parameters correlated with prolonged survival were the initial severity of liver disease, the donor and recipient ages, and the period in which the transplant was performed.

These aspects are not surprising and well documented in previous studies based on very long-term survivals [11, 12, 15, 16].

As regards the state of liver disease, it appears evident that the recipient pre-transplant clinical conditions influence long-term survival. An experience from Los Angeles, US, focused on 168 LT patients surviving at least 20 years after transplant identified the nonurgent LT status as a protective factor for reaching long surviving [15].

A large European experience focused on decompensated cirrhotic patients requiring intensive care hospitalization before LT showed disappointing intent-to-treat 1-year survivals of only 50% in patients with acute-on-chronic liver failure with three or more organs failing [17].

Data from the 2020 OPTN/SRTR annual report confirmed that five-year survival rates among deceased-donor LT recipients exceeded 75 percent in all the categories, except for those over age 65 and with advanced liver disease (i.e., MELD > 40) [18].

In our work, we used the UNOS status to define the pre-transplant clinical condition instead of the MELD score because this latter value was not systematically available in very aged cases. Patients with UNOS 1-2A status had an increased risk of not reaching at least 10 years of follow-up, with an OR = 2.62. Moreover, patients with UNOS status 1-2A had 10- and 20-year survivals of only 30.5 and 28.5%, respectively. Conversely, patients with status 2B-3 showed excellent long-term survival, with 10- and 20-year survival rates of 50.0 and 48.1%, respectively. Looking at the survival curses, it appears evident that patients with a more severe condition (i.e., fulminant hepatic failure or hospitalization in intensive care while waiting for a transplant) show a higher rate of mortality within the first 90 days after transplantation due to their already compromised general conditions. After this initial slump, the distance between the two survival curves stabilizes, indicating that seriously ill patients who can overcome the difficult post-LT period still have good survival prospects. The other aspect to underline is that in our series, half of the patients transplanted in status 2B-3 survive at least 20 years, demonstrating the extraordinary success of the LT procedure in terms of benefit.

Regarding donor age, there is no pre-established limit in the literature above which a graft cannot be considered eligible for LT [19]. Due to organ shortage, there has been a progressive increase over the years in the use of grafts from elderly donors, with cases of nonagenarian donors even reported [20]. The impact of donor age on post-LT mortality has been reported in large international populations [21–23]. A study from the US based on 9,882 donors reported a progressive increase in the risk of post-LT graft failure when the donor age overpassed 40 years (age 40–49: + 17%; 50–59: + 32%; 60–69: + 53%; > 70: + 65%) [21]. A similar study from the Eurotransplant area (N = 5939) confirmed this evidence [22]. An Italian study on 4,207 deceased donors observed a 3% increased risk for liver graft discard for every year of donor age increase [23].

The recipient age has also steadily increased over the past 15 years, with an increased percentage of transplanted patients aged ≥ 70. Also in this case, several studies reported in the literature focused on the worse results reported in this sub-group of cases [24–26].

A large study from Korea (N = 9415) showed that the risk of death among recipients older than 70 years was about four-fold higher compared to patients aged 51–55 years [24]. Similarly, a US study based on the UNOS registry (N = 114,433) reported significantly higher mortality (+ 67%) in patients aged ≥ 70 years when compared to younger patients [25].

A study from Italy (N = 693) focused on LT patients aged ≥ 65 years showed that aged recipients reported more frequent early allograft dysfunction (23.9 vs. 16.8%, P = 0.04), and that recipient age ≥ 65 years was an independent risk factor for patient death (HR = 1.76; P = 0.002) and graft loss (HR = 1.63; P = 0.005) [26].

Based on the data observed in our study, both advanced donor and recipient ages confirmed their negative impact in reaching at least 10 years of follow-up after LT. In detail, the increased risks of not reaching 10 years of follow-up were 1% and 2% for each increased donor and recipient increased year of age, respectively.

Starting from these results, it is relevant to note that while donor and recipient age can influence outcomes, they are just a few of many factors considered during the organ allocation process. Other critical factors include the severity of the recipient’s liver disease, immunological factors, HLA compatibility, and availability of suitable organs.

Consequently, these results must be considered in light of the possible presence of a higher rate of comorbidities in older donors and recipients (i.e., diabetes or arterial hypertension) [27].

Liver transplantation is a complex procedure, and decisions regarding donor and recipient selection should be made on a case-by-case basis, considering the overall risk–benefit ratio for each individual patient. Assessing the biological (more than chronological) age of both the donor and recipient, considering factors such as frailty, comorbidities, and functional status, may provide a better understanding of the overall fitness for transplantation.

As for the LT period, a more than six-fold increased risk of not reaching 10 years of follow-up was observed when the LT was performed in the 1980s. This risk decreased to threefold if the LT was done in the 1990s. Several parameters can justify this phenomenon. Among them are the different surgical techniques adopted [7], the modified indications for transplantation [28], and the evolution in immunosuppression [6].

Interestingly, our study failed to confirm that other parameters were significant predictors of poor long-term follow-up. For example, Duffy et al. [15] and Buescher et al. [16] reported that the female gender was correlated with a higher possibility of overpassing 20 years of post-LT follow-up. In the present study, this evidence was not reported. Because some evidence exists on the negative impact of the donor-to-recipient F–M match [29], we also explored this match. However, also in this case, no statistical relevance was reported in predicting long-term survival.

Another relevant parameter reported in other series was the ischemia time. In detail, Dopazo et al. [12] and Duffy et al. [15] reported a positive correlation between short total ischemia time and long-term survival. In our series, we failed to confirm this datum, in which the total ischemia time was not statistically relevant for the risk of not reaching at least 10 years of post-LT follow-up. Unfortunately, due to the study retrospective nature, we could not analyze the data concerning warm and cold ischemia time in a dichotomized fashion.

The present study has several limits to report. First of all, the study is retrospective, covering a very long time in which several evolutions have been observed in the field of LT. As a consequence, many pieces of information nowadays ordinarily collected (i.e., MELD score, cold and warm ischemia time) were not available in detail in the very early transplanted cases in our database. However, considering the intent to explore these patients principally, we accepted the limitations of the study retrospective nature, trying to minimize the loss of information by only exploring the data available in the entire cohort. Unfortunately, the risk of missing relevant variables not investigable in the present setting is present. Second, the cohort explored is not homogeneous since a significant difference exists among the different historical periods examined. We accepted this limit in light of the necessity to exactly explore these divergences. Third, our study is represented by a monocenter population transplanted in a low-volume center, mostly young donors and recipients (< 60 years) and a relatively short ischemia time. Therefore, the generalizability of our results should be cautiously considered. A larger population involving other centers should improve the validity of our results. Lastly, the analysis has been done only using pre-LT available data, in the absence of an assessment of comorbidities arising in the follow-up, which may have influenced long-term survival. Our intent was only to consider available pre-transplant variables able to influence the long-term survivals. However, collecting data on post-transplant comorbidities should be an interesting approach for adding more information on post-LT management.

## Conclusions

Liver transplantation represents an extraordinary therapy for several severe liver diseases, consenting to reach in half of the transplanted cases even more than 20 years of follow-up. The initial liver function and the donor and recipient ages are relevant in impacting long-term survival after transplantation. A broad commitment from many professional groups, including surgeons, hepatologists, and anesthesiologists, is necessary. The achievement of excellent results in terms of long-term survival proves the effectiveness of this multidisciplinary collaboration.

## Data Availability

The datasets generated during and/or analyzed during the current study are not publicly available but are available from the corresponding author on reasonable request.

## Abbreviations

95.0% CI: 95% confidence intervals
IQR: Interquartile ranges
LASSO: Least absolute shrinkage and selection operator
LS: Long surviving group
LT: Liver transplantation
mTOR: Mammalian target of rapamycin
NASH: Non-alcoholic steatohepatitis
OR: Odds ratio
PDF: Primary dysfunction
PNF: Primary-non-function
SS: Short surviving group
STROBE: Strengthening the reporting of observational studies in epidemiology
UNOS: United network for organ sharing

## References

[1] Rossi M, Mennini G, Lai Q, GinanniCorradini S, Drudi FM, Pugliese F (2007). Liver transplantation. J Ultrasound.

[2] Sapisochin G, Hibi T, Toso C, Man K, Berenguer M, Heimbach J (2021). Transplant oncology in primary and metastatic liver tumors: principles, evidence, and opportunities. Ann Surg.

[3] Starzl TE, Marchioro TL, Vonkaulla KN, Hermann G, Brittain RS, Waddell WR (1963). Homotransplantation of the liver in humans. Surg Gynecol Obstet.

[4] National Institutes of Health Consensus Development Conference Statement (1984). liver transplantation - June 20–23, 1983. Hepatology.

[5] Adam R, Karam V, Cailliez V, Grady JGO, Mirza D, Cherqui D (2018). 2018 annual report of the european liver transplant registry (ELTR) - 50-year evolution of liver transplantation. Transpl Int.

[6] Lerut JP, Pinheiro RS, Lai Q, Stouffs V, Orlando G, Juri JM (2014). Is minimal, [almost] steroid-free immunosuppression a safe approach in adult liver transplantation? Long-term outcome of a prospective, double blind, placebo-controlled, randomized, investigator-driven study. Ann Surg.

[7] Lai Q, Nudo F, Molinaro A, Mennini G, Spoletini G, Melandro F (2011). Does caval reconstruction technique affect early graft function after liver transplantation? A preliminary analysis. Transplant Proc.

[8] Biancofiore G, Bindi M, Ghinolfi D, Lai Q, Bisa M, Esposito M (2017). Octogenarian donors in liver transplantation grant an equivalent perioperative course to ideal young donors. Dig Liver Dis.

[9] Neuberger J, James O (1999). Guidelines for selection of patients for liver transplantation in the era of donor-organ shortage. Lancet.

[10] Roberts MS, Angus DC, Bryce CL, Valenta Z, Weissfeld L (2004). Survival after liver transplantation in the United States: a disease-specific analysis of the UNOS database. Liver Transpl.

[11] Waki K (2006) UNOS liver registry: ten year survivals. Clin Transpl 29–39 18368704

[12] Dopazo C, Bilbao I, Castells LL, Sapisochin G, Moreiras C, Campos-Varela I (2015). Analysis of adult 20-year survivors after liver transplantation. Hepatol Int.

[13] Institute of Medicine (US) Committee on Organ Procurement and Transplantation Policy (1999). Organ procurement and transplantation: assessing current policies and the potential impact of the DHHS final rule.

[14] Rizzetto M, Macagno S, Chiaberge E, Verme G, Negro F, Marinucci G (1987). Liver transplantation in hepatitis delta virus disease. Lancet.

[15] Duffy JP, Kao K, Ko CY, Farmer DG, McDiarmid SV, Hong JC (2010). Long-term patient outcome and quality of life after liver transplantation: analysis of 20-year survivors. Ann Surg.

[16] Buescher N, Seehofer D, Helbig M, Andreou A, Bahra M, Pascher A (2016). Evaluating twenty-years of follow-up after orthotopic liver transplantation, best practice for donor-recipient matching: what can we learn from the past era?. World J Transplant.

[17] Belli LS, Duvoux C, Artzner T, Bernal W, Conti S, Cortesi PA, ELITA/EF-CLIF working group (2021). Liver transplantation for patients with acute-on-chronic liver failure (ACLF) in Europe: results of the ELITA/EF-CLIF collaborative study (ECLIS). J Hepatol.

[18] Kwong AJ, Ebel NH, Kim WR, Lake JR, Smith JM, Schladt DP (2022). OPTN/SRTR 2020 annual data report: liver. Am J Transplant.

[19] Ghinolfi D, Lai Q, Pezzati D, De Simone P, Rreka E, Filipponi F (2018). Use of elderly donors in liver transplantation: a paired-match analysis at a single center. Ann Surg.

[20] Ghinolfi D, Pezzati D, Rreka E, Balzano E, Catalano G, Coletti L (2019). Nonagenarian grafts for liver transplantation. Liver Transpl.

[21] Feng S, Goodrich NP, Bragg-Gresham JL, Dykstra DM, Punch JD, DebRoy MA (2006). Characteristics associated with liver graft failure: the concept of a donor risk index. Am J Transplant.

[22] Braat AE, Blok JJ, Putter H, Adam R, Burroughs AK, Rahmel AO, European Liver and Intestine Transplant Association (ELITA) and Eurotransplant Liver Intestine Advisory Committee (ELIAC) (2012). The Eurotransplant donor risk index in liver transplantation: ET-DRI. Am J Transplant.

[23] Lai Q, Ghinolfi D, Avolio AW, Manzia TM, Mennini G, Melandro F (2022). Proposal and validation of a liver graft discard score for liver transplantation from deceased donors: a multicenter Italian study. Updates Surg.

[24] Gil E, Kim JM, Jeon K, Park H, Kang D, Cho J (2018). Recipient age and mortality after liver transplantation: a population-based cohort study. Transplantation.

[25] Sharma M, Ahmed A, Wong RJ (2017). Significantly higher mortality following liver transplantation among patients aged 70 years and older. Prog Transplant.

[26] Melandro F, Lai Q, Ghinolfi D, Manzia TM, Spoletini G, Rossi M (2023). Outcome of liver transplantation in elderly patients: an Italian multicenter case-control study. Updates Surg.

[27] Bhat V, Tazari M, Watt KD, Bhat M (2018). New-onset diabetes and preexisting diabetes are associated with comparable reduction in long-term survival after liver transplant: a machine learning approach. Mayo Clin Proc.

[28] Manzia TM, Trapani S, Nardi A, Ricci A, Lenci I, Milana M (2022). Temporal trends of waitlistings for liver transplantation in Italy: the ECALITA (Evolution of IndiCAtion in LIver transplantation in ITAly) registry study. Dig Liver Dis.

[29] Lai Q, Giovanardi F, Melandro F, LarghiLaureiro Z, Merli M, Lattanzi B (2018). Donor-to-recipient gender match in liver transplantation: a systematic review and meta-analysis. World J Gastroenterol.

